# Influence of genetic variants of opioid-related genes on opioid-induced adverse effects in patients with lung cancer: A STROBE-compliant observational study: Erratum

**DOI:** 10.1097/MD.0000000000028405

**Published:** 2021-12-23

In the article, “Influence of genetic variants of opioid-related genes on opioid-induced adverse effects in patients with lung cancer: A STROBE-compliant observational study”^[1]^ which published in Volume 100, Issue 44 of *Medicine*¸ the values in the OR column of Table 2 were rounded. The correct table appears below and have been corrected in the published article.

Insert Table 2 

**Table:** 

	rs1799971 Alt allele freq: 0.494 A > G *ORRM1*										rs2234918 Alt allele freq: 0.79 C > T *OPRD1*									
Allele	A/A	A/G + G/G	OR	95% CI	*P*	G/G	A/A + A/G	OR	95% CI	*P*	C/C	C/T + T/T	OR	95% CI	*P*	T/T	C/C + C/T	OR	95% CI	*P*
	n = 22	n = 66				n = 21	n = 67				n = 5	n = 83				n = 56	n = 32			
Somnolence	6 (27.3%)	13 (19.7%)	1.5	0.5 to 4.7	.55^∗^	0 (0.0%)	19 (28.4%)	0	0 to 0.6	.005^∗^	1 (20.0%)	18 (21.7%)	0.9	0.1 to 8.6	1.00^∗^	10 (17.9%)	9 (28.1%)	0.6	0.2 to 1.6	.29^∗^
Nausea	1 (4.5%)	13 (19.7%)	0.2	0.02 to 1.6	.17^∗^	5 (23.8%)	9 (13.4%)	2.0	0.6 to 6.9	.31^∗^	0 (0.0%)	14 (16.9%)	0	0 to 6.0	1.00^∗^	12 (21.4%)	2 (6.3%)	4.1	0.9 to 19.6	.07^∗^
Constipation	10 (45.5%)	24 (36.4%)	1.5	0.6 to 3.9	.46^∗^	9 (42.9%)	25 (37.3%)	1.3	0.5 to 3.4	.49^∗^	1 (20.0%)	33 (39.8%)	0.4	0.04 to 3.5	.64^∗^	23 (41.1%)	11 (34.4%)	1.3	0.5 to 3.3	.65^∗^
Delirium	1 (4.5%)	9 (13.6%)	0.3	0.04 to 2.5	.44^∗^	3 (14.2%)	7 (10.4%)	1.4	0.3 to 6.1	.70^∗^	0 (0.0%)	10 (12.0%)	0	0 to 9.2	1.00^∗^	6 (10.7%)	4 (12.5%)	0.8	0.2 to 3.2	1.00^∗^
Urinary retention	0 (0.0%)	3 (4.5%)	0	0 to 7.6	1.00^∗^	2 (9.5%)	1 (1.5%)	7.0	0.6 to 80.8	.14^∗^	0 (0.0%)	3 (3.6%)	0	0 to 46.4	1.00^∗^	2 (3.6%)	1 (3.1%)	1.2	0.1 to 13.2	1.00^∗^
Pruritus	4 (18.1%)	8 (12.1%)	1.6	0.5 to 6.0	.49^∗^	2 (9.5%)	10 (14.9%)	0.6	0.1 to 3.00	.72^∗^	0 (0.0%)	12 (14.5%)	0	0 to 7.3	1.00^∗^	8 (14.3%)	4 (12.5%)	1.2	0.3 to 4.2	1.00^∗^

	rs1051660 Alt allele freq: 0.146 C > A *OPRK1*										rs4680 Alt allele freq: 0.335 G > A *COMT*									
Allele	C/C	C/A + A/A	OR	95% CI	*P*	A/A	C/C + C/A	OR	95% CI	*P*	G/G	G/A + A/A	OR	95% CI	*P*	A/A	G/G + G/A	OR	95% CI	*P*
	n = 60	n = 22				n = 2	n = 80				n = 40	n = 48				n = 11	n = 77			
Somnolence	15 (25.0%)	3 (13.6%)	2.1	0.6 to 8.2	.37^∗^	1 (50.0%)	17 (21.3%)	3.7	0.2 to 62.4	.39^∗^	14 (35.0%)	5 (10.4%)	4.5	1.4 to 18.1	.008^∗^	0 (0%)	19 (24.7%)	0	0 to 1.4	.11^∗^
Nausea	10 (16.7%)	4 (18.2%)	0.9	0.3 to 3.2	1.00^∗^	1 (50.0%)	13 (16.3%)	5.2	0.3 to 87.8	.31^∗^	8 (20.0%)	6 (12.5%)	1.8	0.6 to 5.6	.39^∗^	0 (0%)	14 (18.2%)	0	0 to 2.4	.36^∗^
Constipation	21 (35.0%)	10 (45.5%)	0.7	0.2 to 1.7	.45^∗^	1 (50.0%)	30 (37.5%)	1.7	0.1 to 27.6	1.00^∗^	17 (42.5%)	17 (35.4%)	1.4	0.6 to 3.2	.51^∗^	3 (27.3%)	31 (40.3%)	0.6	0.1 to 2.3	.52^∗^
Delirium	7 (11.7%)	3 (13.6%)	0.8	0.2 to 3.6	1.00^∗^	0 (0.0%)	10 (12.5%)	0	0 to 39.7	1.00^∗^	3 (7.5%)	7 (14.6%)	0.5	0.1 to 2.0	.34^∗^	2 (18.2%)	8 (10.4%)	1.9	0.4 to 10.5	.61^∗^
Urinary retention	2 (3.3%)	1 (4.5%)	0.7	0.1 to 8.4	1.00^∗^	0 (0.0%)	3 (3.8%)	0	0 to 146.8	1.00^∗^	1 (2.5%)	2 (4.2%)	0.6	0.1 to 6.8	1.00^∗^	0 (0%)	3 (3.9%)	0	0 to 17.8	1.00^∗^
Pruritus	8 (13.3%)	2 (9.1%)	1.5	0.3 to 7.9	.72^∗^	0 (0.0%)	10 (12.5%)	0	0 to 39.7	1.00^∗^	6 (15.0%)	6 (12.5%)	1.2	0.4 to 4.2	.76^∗^	0 (0%)	12 (15.6%)	0	0 to 2.5	1.00^∗^

	rs6275 Alt allele freq: 0.352 A > G *DRD2*										rs1045642 Alt allele freq: 0.534 A > G *ABCB1*									
Allele	A/A	A/G + G/G	OR	95% CI	*P*	G/G	A/A + A/G	OR	95% CI	*P*	A/A	A/G or G/G	OR	95% CI	*P*	G/G	A/A + A/G	OR	95% CI	*P*
	n = 40	n = 45				n = 15	n = 70				n = 23	n = 65				n = 29	n = 59			
Somnolence	9 (22.5%)	10 (22.2%)	1.0	0.4 to 2.8	1.00^∗^	3 (20.0%)	16 (22.9%)	0.8	0.2 to 3.4	1.00^∗^	8 (34.8%)	11 (16.9%)	2.6	0.9 to 7.7	.08^∗^	4 (13.8%)	15 (25.4%)	0.5	0.1 to 1.6	.28^∗^
Nausea	8 (20.0%)	5 (11.1%)	2.0	0.6 to 6.7	.37^∗^	1 (6.7%)	12 (17.1%)	0.4	0.04 to 2.9	.45^∗^	4 (17.4%)	10 (15.4%)	1.2	0.3 to 4.1	.75^∗^	4 (13.8%)	10 (16.9%)	0.8	0.2 to 2.8	1.00^∗^
Constipation	15 (37.5%)	19 (42.2%)	0.8	0.3 to 2.0	.82^∗^	9 (60.0%)	25 (35.7%)	2.7	0.9 to 8.5	.09^∗^	12 (52.2%)	22 (33.8%)	2.1	0.8 to 5.6	.14^∗^	11 (37.9%)	23 (39.0%)	1.0	0.4 to 2.4	1.00^∗^
Delirium	6 (15.0%)	4 (8.9%)	1.8	0.5 to 6.9	.51^∗^	1 (6.7%)	9 (12.9%)	0.5	0.1 to 4.1	.68^∗^	1 (4.3%)	9 (13.8%)	0.3	0.03 to 2.4	.44^∗^	5 (17.2%)	5 (8.5%)	2.3	0.6 to 8.5	.29^∗^
Urinary retention	1 (2.5%)	2 (4.4%)	0.6	0.1 to 6.3	1.00^∗^	1 (6.7%)	2 (2.9%)	2.4	0.2 to 28.7	.45^∗^	0 (0.0%)	3 (4.6%)	0	0 to 6.9	.56^∗^	2 (6.9%)	1 (1.7%)	4.3	0.4 to 49.5	.25^∗^
Pruritus	4 (10.0%)	7 (15.6%)	0.6	0.2 to 2.2	.53^∗^	1 (6.7%)	10 (14.3%)	0.4	0.1 to 3.6	.68^∗^	5 (21.7%)	7 (10.8%)	2.3	0.7 to 8.1	.29^∗^	3 (10.3%)	9 (15.3%)	0.6	0.2 to 2.6	.74^∗^

	rs2070995 Alt allele freq: 0.642 T > C *GIRK2*										rs324420 Alt allele freq: 0.138 C > A *FAAH*									
Allele	T/T	C/C + C/T	OR	95% CI	*P*	C/C	C/A + A/A	OR	95% CI	*P*	C/C	C/A + A/A	OR	95% CI	*P*	A/A	C/C + C/A	OR	95% CI	*P*
	n = 10	n = 78				n = 35	n = 53				n = 67	n = 20				n = 4	n = 83			
Somnolence	2 (20.0%)	17 (21.8%)	0.9	0.2 to 4.6	1.00^∗^	9 (25.7%)	10 (18.9%)	1.5	0.5 to 4.1	.60^∗^	15 (22.4%)	4 (20.0%)	1.2	0.3 to 4.0	1.00^∗^	0 (0.0%)	19 (22.9%)	0	0 to 5.5	.57^∗^
Nausea	2 (20.0%)	12 (15.4%)	1.4	0.3 to 7.3	.66^∗^	7 (20.0%)	7 (13.2%)	1.6	0.5 to 5.2	.55^∗^	11 (16.4%)	3 (15.0%)	1.1	0.3 to 4.5	1.00^∗^	1 (25.0%)	13 (15.7%)	1.8	0.2 to 18.6	.51^∗^
Constipation	4 (40.0%)	30 (38.5%)	1.1	0.3 to 4.1	1.00^∗^	18 (51.4%)	16 (30.2%)	2.5	1.0 to 5.9	.07^∗^	25 (37.3%)	9 (45.0%)	0.7	0.3 to 1.0	.61^∗^	2 (50.0%)	32 (38.6%)	1.6	0.2 to 11.9	.64^∗^
Delirium	1 (10.0%)	9 (11.5%)	0.9	0.1 to 7.5	1.00^∗^	4 (11.4%)	6 (11.3%)	1.0	0.3 to 3.9	1.00^∗^	6 (9.0%)	4 (20.0%)	0.4	0.1 to 1.6	.23^∗^	0 (0.0%)	10 (12.0%)	0	0 to 12.5	1.00^∗^
Urinary retention	0 (0.0%)	3 (3.8%)	0	0 to 20.0	1.00^∗^	2 (5.7%)	1 (1.9%)	3.2	0.3 to 36.2	.56^∗^	3 (4.5%)	0 (0.0%)	∞	0.1 to ∞	1.00^∗^	0 (0.0%)	3 (3.6%)	0	0 to 61.0	1.00^∗^
Pruritus	1 (10.0%)	11 (14.1%)	0.7	0.8 to 5.9	1.00^∗^	6 (17.1%)	6 (11.3%)	1.6	0.5 to 5.5	.53^∗^	8 (11.9%)	4 (20.0%)	0.5	0.1 to 2.0	.46^∗^	1 (25.0%)	11 (13.3%)	2.2	0.2 to 22.9	.45^∗^

∗ Fisher's exact test *ABCB1* = Adenosine Triphosphate Binding Cassette B1, CI = Confidence Interval, *COMT* = Catechol-O-Methyltransferase, *DRD2* = Dopamine Receptor D2, *FAAH* = Fatty Acid Amide Hydrolase, *GIRK2* = G-protein regulated Inward Rectifier potassium channel 2, *OPRD1* = Opioid δ receptor 1, *OPRK1* = Opioid κ Receptor 1, *OPRM1* = Opioid μ Receptor 1, OR = Odds Ratio
